# Development of practice and consensus-based strategies including a treat-to-target approach for the management of moderate and severe juvenile dermatomyositis in Germany and Austria

**DOI:** 10.1186/s12969-018-0257-6

**Published:** 2018-06-25

**Authors:** Claas H. Hinze, Prasad T. Oommen, Frank Dressler, Andreas Urban, Frank Weller-Heinemann, Fabian Speth, Elke Lainka, Jürgen Brunner, Heike Fesq, Dirk Foell, Wolfgang Müller-Felber, Ulrich Neudorf, Christoph Rietschel, Tobias Schwarz, Ulrike Schara, Johannes-Peter Haas

**Affiliations:** 1 Department of Pediatric Rheumatology and Immunology, University Hospital Münster, Albert-Schweitzer-Campus 1, Building D3, 48149 Münster, Germany; 2 Department of Pediatric Oncology, Hematology and Clinical Immunology, University Hospital Düsseldorf, Düsseldorf, Germany; 3 Department of Pediatric Pulmonology, Allergy and Neonatology, Hanover Medical School, Hanover, Germany; 4 Department of Pediatrics, St. Mary’s Hospital, Amberg, Germany; 5 Division of Pediatric Rheumatology, Prof. Hess Children’s Hospital, Bremen, Germany; 6 Division of Pediatric Rheumatology, University Medicine, Rostock, Germany; 7 Division of Immunology, Bone Marrow Transplantation and Rheumatology, University Hospital Ulm, Ulm, Germany; 8 Department of Pediatrics, University Hospital Essen, Essen, Germany; 9 Department of Pediatrics, Medical University Innsbruck, Innsbruck, Austria; 10 German Center for Pediatric and Adolescent Rheumatology, Garmisch-Partenkirchen Department of Dermatology, Oberammergau Center for Rheumatic Diseases, Oberammergau, Germany; 11 Department of Pediatric Neurology, University Hospital Munich, Munich, Germany; 12 Department of Pediatrics, Clementine Children’s Hospital, Frankfurt, Germany; 13 Department of Pediatric Rheumatology, St. Josef Hospital, Sendenhorst, Germany; 14 Department of Pediatric Neurology, University Hospital Essen, Essen, Germany

**Keywords:** Dermatomyositis, Child, Consensus, Diagnosis, Antirheumatic agents, Comparative effectiveness research

## Abstract

**Background:**

Juvenile dermatomyositis (JDM) is the most common inflammatory myopathy in childhood and a major cause of morbidity among children with pediatric rheumatic diseases. The management of JDM is very heterogeneous. The JDM working group of the Society for Pediatric Rheumatology (GKJR) aims to define consensus- and practice-based strategies in order to harmonize diagnosis, treatment and monitoring of JDM.

**Methods:**

The JDM working group was established in 2015 consisting of 23 pediatric rheumatologists, pediatric neurologists and dermatologists with expertise in the management of JDM. Current practice patterns of management in JDM had previously been identified via an online survey among pediatric rheumatologists and neurologists. Using a consensus process consisting of online surveys and a face-to-face consensus conference statements were defined regarding the diagnosis, treatment and monitoring of JDM. During the conference consensus was achieved via nominal group technique. Voting took place using an electronic audience response system, and at least 80% consensus was required for individual statements.

**Results:**

Overall 10 individual statements were developed, finally reaching a consensus of 92 to 100% regarding (1) establishing a diagnosis, (2) case definitions for the application of the strategies (moderate and severe JDM), (3) initial diagnostic testing, (4) monitoring and documentation, (5) treatment targets within the context of a treat-to-target strategy, (6) supportive therapies, (7) explicit definition of a treat-to-target strategy, (8) various glucocorticoid regimens, including intermittent intravenous methylprednisolone pulse and high-dose oral glucocorticoid therapies with tapering, (9) initial glucocorticoid-sparing therapy and (10) management of refractory disease.

**Conclusion:**

Using a consensus process among JDM experts, statements regarding the management of JDM were defined. These statements and the strategies aid in the management of patients with moderate and severe JDM.

## Background

Juvenile dermatomyositis (JDM) is the most common inflammatory myopathy of childhood and a major cause of morbidity and mortality among patients with pediatric rheumatic diseases [1]. The management of JDM is highly variable internationally [2, 3]. Few controlled trials have been performed on the treatment of JDM, e.g. on methotrexate (MTX), cyclosporin A (CSA) and rituximab (RTX) [4, 5]. The type of glucocorticoid regimen used is especially variable, with many centers using high-dose oral glucocorticoid therapy while others use intermittent intravenous methylprednisolone pulse therapy [6–10]. Multiple smaller open label or retrospective studies have been performed, e.g. on cyclophosphamide (CYC), tumor necrosis factor inhibitors (TNFi), high-dose intravenous immune globulins (IVIG), or mycophenolate mofetil (MMF) [9, 11–13]. Frequently, treatment is based on expert opinion [1, 6, 14]. Notably, only few formally approved treatment options exist for the treatment of dermatomyositis (DM), in Germany, including various glucocorticoids (methylprednisolone, prednisolone, prednisone, dexamethasone, triamcinolone), azathioprin [AZA], and, under certain conditions, IVIG) [15]. However, none of these medications have been specifically approved for the use in JDM. Recently, consensus treatment plans have been established in North America by the Childhood Arthritis & Rheumatology Research Alliance (CARRA) and consensus-based treatment recommendations were developed by the Single Hub and Access point for pediatric Rheumatology in Europe (SHARE) initiative [16, 17]. The role of biologic agents in the management of JDM is less well defined, even though they are frequently used as outlined by a recent survey among CARRA members [18]. However, there is evidence that treatment approaches for JDM in Germany vary in several aspects from those used in other countries, including the choice of glucocorticoid therapy regimens [19]. Lately, the concept of treat-to-target and tight control has been a cornerstone of current treatment recommendations for rheumatoid arthritis, systemic lupus erythematosus and gout, and has been discussed for pediatric rheumatic diseases as well [20–24].

The PRO-KIND (PROjekte zur Klassifikation, Überwachung und Therapie in der KINDerrheumatologie; projects for the classification, monitoring and therapy in pediatric rheumatology) initiative is a sub-committee of the Society for Pediatric Rheumatology (GKJR) in Germany and Austria and aims to define consensus-based plans to harmonize diagnostic and treatment approaches. This initiative was started since it was perceived that children with juvenile rheumatic diseases in Germany and Austria often are either treated too late or not treated with the most up-to-date therapeutic regimens. To meet this challenge, the goal of the PRO-KIND initiative is to foster the use of harmonized diagnostic and treatment plans with defined targets [25, 26]. Previously, the JDM working group had identified current practice patterns in Germany and Austria among pediatric rheumatologists and neurologists [19].

As a next step, the goal of the PRO-KIND working group on JDM was to harmonize previously identified patterns into plans and statements.

## Methods

### PRO-KIND JDM working group and expert panel

The JDM working group was formed in April 2015, initially comprised of 12 pediatric rheumatologists with clinical expertise in the management of patients with JDM. Identification of project goals took place via a formal online survey using the web-based program SurveyMonkey (SurveyMonkey Inc.; San Mateo, California, USA; www.surveymonkey.com). Nine pediatric rheumatologists participated in an initial face-to-face meeting in January 2016. There was consensus that the expert panel should also include expert pediatric neurologists. Therefore, 2 pediatric neurologists with special expertise in managing JDM (W. M-F. and U.S.) were also invited to join the group, forming an extended interdisciplinary expert panel.

### Consensus conference

An extended expert panel with 13 voting members (11 pediatric rheumatologists, 2 pediatric neurologists) and a non-voting dermatologist experienced in the care of children with JDM participated in a final face-to-face consensus meeting on January 13, 2017. The extended expert panel overlapped with but was not identical with the members of the initial working group. Prior to the consensus conference, the voting members had received access to various essential data via sciebo, a cloud service hosted by the state of Northrhine-Westphalia. The data provided included detailed results of previous online surveys, previous meeting protocols, essential literature obtained via an extensive literature search (but not formally graded), including the 112 papers that were scored by SHARE [16]. The process was guided by a psychologist trained as a professional moderator. We used nominal group technique for consensus building. For each individual statement, the following procedures were performed: the statement and its background were presented by one of the co-authors (C.H.) who had formulated the statement in question. Subsequently, every participant of the consensus conference had 1 min of time available to raise issues with the statement being discussed. These issues were recorded on a flip-chart. Then there was a 15-min open discussion of these items to further improve the statement. Changes to the statement were made ad hoc by another co-author (P.O.) using the PowerPoint presentation program, visible to all participants. Finally, anonymous voting took place during which each participant could either accept or reject the respective statement via an electronic audience response system. Consensus was considered to be present if at least 80% of experts supported a statement.

## Results

### Expert panel

The expert status of the panel members is demonstrated by the fact that centers represented in the panel contributed more than half of all patients (for example, in 2016: 72 out of 127 [57%]) with JDM documented within the National Pediatric Rheumatic Disease Database (“Kerndokumentation rheumakranker Kinder und Jugendlicher”).

### Diagnosis and case definition

Regarding the diagnosis of JDM, there was consensus that the conventional Bohan and Peter criteria should be modified. In particular, there was consensus that typical magnetic resonance imaging (MRI) findings represented an important finding for establishing a diagnosis of JDM. Furthermore, electromyography was removed from the list of findings. The finding of symmetric proximal muscle weakness was modified to also include myalgia as a possible criterion (Table 1). Concerning the statements developed and the resulting treatment strategies, the group devised a case definition for moderate and severe JDM, similar to an existing CARRA definition (Table 1) [17]. According to the consensus, all patients with active moderate or severe JDM may be treated with strategies outlined here. Regarding this manuscript, we will from now on simply refer to JDM, indicating moderate or severe JDM. Additionally, the group developed a list of diagnostic tests that may be useful in patients with JDM. While some of the tests may be useful for establishing a diagnosis of JDM, others may be useful to rule out alternative diagnoses or to assess for potential organ involvement or complications. Also, myositis-specific antibodies were felt to be an important aspect of the work-up.

**Table 1:** Diagnosis of juvenile dermatomyositis and case definitions

Statements	Consensus
Diagnosis	100%
For the diagnosis of juvenile dermatomyositis, the following findings should be present prior to age 18 years: • Typical skin finding (heliotrope and/or Gottron-sign/−papules) Additional criteria: • Symmetric proximal muscle weakness and/or myalgia • Increased muscle-related enzymes (creatine kinase, glutamate oxaloacetate transaminase, lactate dehydrogenase and/or aldolase) • Typical findings on muscle biopsy • Typical findings on magnetic resonance imaging Other possible etiologies should be excluded. Probable JDM: Skin findings and at least 2 additional criteria Definite JDM: Skin findings and at least 3 additional criteria	
Case definition	92%
The treatment strategies discussed below apply to patients with active moderately severe or severe juvenile dermatomyositis. Patients with severe juvenile dermatomyositis fulfill at least one of the following characteristics and none of these characteristics is present in patients with moderate JDM: • Age < 1 year • Requirement for intensive care therapy • Marked disability as measured by being bedridden, childhood myositis assessment scale (CMAS) < 15 or manual muscle testing (MMT)8 < 30 • Relevant pulmonary disease • Myocarditis • Vasculitis with organ involvement (gastrointestinal tract, kidney, lungs or central nervous system) • Aspiration or marked dysphagia • Severe cutaneous ulceration • Significant calcinosis	
Diagnostic work-up	100%
Obtaining the following parameters may be useful in case of probable or definite JDM: • Laboratory tests: o Enzymes (creatine kinase, glutamate oxaloacetate transaminase, glutamate pyruvate transaminase, lactate dehydrogenase, aldolase) o Complete blood count with differential count o Routine serum chemistry panel o Inflammatory markers (C-reactive protein, erythrocyte sedimentation rate) o Antinuclear antibody (ANA) o In case of positive ANA: anti-ds-DNA, anti-PM-Scl, Sm antibodies, anti-SS-A, anti-SS-B and anti-U1RNP antibodies o Extended myositis-blot/myositis-specific antibody (incl. Anti-synthetase, anti-MDA5, anti-Mi-2, anti-NXP-2, anti-SRP, anti-TIF-1-γ antibodies) o Immunoglobulin (Ig)G, IgA, IgM o Complement (CH50) o Von Willebrand factor-antigen o Troponin o TSH o Immunization status o Urinalysis o Stool for occult blood • Further testing: o Electrocardiography o Echocardiography o Magnetic resonance imaging (incl. Short tau inversion recovery or equivalent sequences) o Chest X-ray o Abdominal ultrasound o Muscle ultrasound o Muscle biopsy o Bodyplethysmography with CO-diffusion capacity o Capillary microscopy	

### Disease monitoring

It was felt to be important to define a minimum dataset that should be routinely collected on all patients with at least moderate JDM (Table 2). The group discussed the various validated parameters, taking into account the familiarity of practitioners in Germany with the respective investigations based on a recently performed online survey among pediatric rheumatologists and neurologists in Germany [16, 19, 27–30]. The investigations include measurements of global, muscular and extramuscular disease activity, functional impairment, quality of life and disease damage.

**Table 2:** Monitoring parameters for patients with juvenile dermatomyositis

Statement	Consensus
Disease monitoring tools	100%
In order to monitor disease activity and disease damage over time, the regular measurement of the following parameters may be useful (initially every 6 weeks, later every 3 months): • Length, weight, blood pressure • Physician global assessment of disease activity (visual analog scale 0–10) • Parent/Patient global assessment of disease activity (visual analog 0–10) • Manual muscle testing (MMT)8 and/or Childhood Myositis Assessment Scale (CMAS) • Childhood Health Assessment Questionnaire (CHAQ) • A validated Quality of life instrument • Muscle enzymes • Extramuscular disease activity, e.g. via Disease Activity Score, Myositis Disease Activity Assessment Tool or Visual Analog Scale • Myositis damage index (annually)	

### Treatment strategy and treatment targets

The group defined a treat-to-target strategy, which means treatment to be adjusted according to the achievement of previously defined targets (Table 3). It was felt that generally, the initial phase of therapy should be more intensive in order to quickly achieve improvement (“remission induction”) and avoid undertreatment, with the aim to reduce damage, such as contractures, calcinosis and chronic muscle weakness. The reason for this notion was two-fold: first, there is a high risk of complications in the acute phase of the disease, and, second, there is an increased risk for disease damage with longer duration of active disease [31–34]. Concerning treatment targets, there was consensus that at least a moderate improvement should be observed within 6 weeks after implementing a major change in therapy and at least a major improvement within 12 weeks after a major change in therapy, with improvement defined according to the American College of Rheumatology/European League Against Rheumatism criteria (Table 3) [35]. The consensus on the 12-month goal was to achieve a glucocorticoid-free treatment regimen. This is similar to what the CARRA consensus treatment plans state [17].

**Table 3:** Treatment targets and treatment strategy

Statements	Consensus
Treatment targets	100%
The overall goal is clinical inactive disease within 1 year after initiation of therapy, ideally under a glucocorticoid-free treatment regimen. Under some circumstances, low-dose glucocorticoids or intermittent intravenous methylprednisolone pulse therapy may be acceptable. The following interim improvement^a^ is targeted: • At least a moderate improvement within 6 weeks after initiation or substantial change in therapy. • At least a major improvement within 3 months after initiation or substantial change in therapy.	
General treatment strategy	92%
The consensus treatment strategies for JDM serve to harmonize existing therapies in clinical practice. The treatment strategy generally consists of a treat-to-target strategy, i.e. therapies are modified according to reaching or failing previously established targets. In addition, there is a more intensive first (induction) treatment phase (6–8 weeks) and a less intensive subsequent (maintenance) phase. Components of the initial therapy include glucocorticoids and glucocorticoid-sparing DMARDs.	

^a^ American College of Rheumatology/European League Against Rheumatism criteria (categories: no, minimal, moderate, major improvement) [34]

### Specific therapies for juvenile dermatomyositis

#### Supportive therapies

There was consensus that all patients with JDM should receive supportive therapy including physical therapy to prevent contractures, muscle weakness, de-conditioning and disability. Early return to sports activities is encouraged as long as it is safely possible. Effective sun and/or ultraviolet light protection using avoidance, textile protection or sunscreen is strongly suggested. Additional supportive therapies may include hydroxychloroquin (HCQ), vitamin D or calcium supplementation (Table 4). It was debated whether HCQ constituted a supportive therapy or a disease-modifying antirheumatic drug (DMARD) in the context of JDM, i.e. a medication that may fundamentally alter the course of disease, in the context of JDM. Eventually, there was consensus that there is not enough evidence to consider HCQ a DMARD sensu stricto. Therefore, in order to avoid the (mis)use of HCQ in case of refractory disease as a means to intensify therapy, it was opted to define HCQ as a supportive agent for the purpose of this project.

**Table 4:** Therapies used in juvenile dermatomyositis

Statements	Consensus
Supportive therapy	100%
Early and intensive physical therapy is essential in order to avoid contractures and improve/maintain muscle strength, even during active myositis. Participation in sports is desirable after individual counseling. Effective sun protection is essential, incl. textile protection and sunscreen. The following treatments may be considered individually: • Supplementation of vitamin D, e.g. depending on 25-OH vitamin D level, glucocorticoid treatment and/or bone mineral density • Supplementation of calcium, e.g. in case of insufficient intake • Hydroxychloroquin	
Initial glucocorticoid therapy	92%
The principal glucocorticoid strategy options include • Intermittent intravenous methylprednisolone pulse (IVMP) therapy + moderate-to-high-dose daily glucocorticoids (prednisone equivalent 0.5–2 mg/kg [max. 80 mg] daily) • Intermittent IVMP therapy + lower-to-moderate dose daily glucocorticoids (prednisone equivalent 0.2–0.5 mg/kg daily) • High-dose daily glucocorticoid therapy (2 mg/kg [max. 60–80 mg] daily) +/− initial single IVMP pulse	
Glucocorticoid tapering	92%
The following landmarks may be applied when tapering glucocorticoids, assuming an adequate treatment response (i.e. treatment targets are reached): • IVMP o 20–30 mg/kg (max. 1000 mg) daily × 3 days o At 2 and 4 weeks, subsequently every 4–6 weeks × 6–12 months • High-dose daily glucocorticoids (prednisone or prednisolone) o 2 mg/kg (max. 60–80 mg) × 1 month, then start taper o 50% of initial dose at 2 months, 25% at 4 months, 12.5% at 6 months, discontinue at 12 months • Moderate-dose daily glucocorticoids (0.5–2 mg/kg daily, < 60 mg daily) o Stop within 12 months • Lower-dose daily glucocorticoids (< 0.5 mg/kg daily) o Stop within 12 months	
Glucocorticoid-sparing therapies	100%
The following glucocorticoid-sparing therapies are used in the initial treatment: • Methotrexate (MTX) 15–20 mg/m^2^ (max. 30 mg) once weekly (s.c. preferred) • MTX 15–20 mg/m^2^ (max. 30 mg) once weekly (s.c. preferred) + intravenous immune globulins (IVIG) (especially in case of severe juvenile dermatomyositis) In case of MTX intolerance, MTX can be replaced by azathioprin (AZA), cyclosporin A (CSA) or mycophenolate mofetil (MMF).	
Refractory disease	92%
In case of not reaching predefined targets (Table 3) or disease flare, a change in therapy is required. In this situation, current therapies should be intensified, exchanged and/or another therapy added. Further treatment should be discussed individually with experts. The following agents are generally used: AZA, calcineurin inhibitors, cyclophosphamide, IVIG, MMFmycophenolate mofetil, rituximab, tumor necrosis factor inhibitors	

*Abbreviations*: *AZA* azathioprin, *CSA* cyclosporin A, *IVIG* intravenous immune globulins, *IVMP* intravenous methylprednisolone pulse, *JDM* juvenile dermatomyositis, *MTX* methotrexate, *MMF* mycophenolate mofetil, *s.c.* subcutaneously

#### Glucocorticoid therapies

Different glucocorticoid regimens were preferred by experts within the panel. Based on the results of a previous survey [19], 3 glucocorticoid regimens have been listed which were generally acceptable to the group members in the treatment of patients with new-onset JDM. The regimens included an intermittent intravenous methylprednisolone pulse (IVMP) therapy in combination with either low-to-moderate (prednisone/prednisolone [PDN] equivalent 0.2–0.5 mg/kg/day) or moderate-to-high-dose (PDN equivalent 0.5–2 mg/kg max. 60–80 mg/day) glucocorticoids, or conventional high-dose oral glucocorticoids (PDN equivalent 2 mg/kg max. 60–80 mg/day) with or without a single IVMP pulse (Table 4).

#### Tapering of glucocorticoids

No consensus was found concerning a specific tapering protocol for glucocorticoids. However, the group defined landmarks for the glucocorticoid taper, i.e. the aim to discontinue glucocorticoids after 12 months of therapy, and, in case of initial high-dose oral glucocorticoid therapy, PDN doses of 50, 25 and 12.5% 2 months, 4 months and 6 months after initiation of therapy, respectively (Table 4).

#### Choice of initial disease-modifying antirheumatic drug therapy

There was consensus that all patients with at least moderate JDM should receive MTX therapy, preferentially subcutaneously, or, if there was intolerance to MTX, an alternative disease-modifying antirheumatic drug (DMARD) therapy, such as AZA, CSA or MMF. Furthermore, there was consensus that high-dose IVIG may be used in conjunction with DMARD therapy, especially in case of severe JDM (Table 4). However, specific regimens of IVIG therapy were not addressed.

#### Treatment of refractory disease

The expert group considered refractory disease to be present if the predefined treatment targets were not reached. Furthermore, patients with longstanding (i.e. not new-onset) JDM may be treated according to strategies outlined here. The group considered two options to be viable under these circumstances: (1) Additional therapy with another DMARD, specifically, AZA, calcineurin inhibitos (CSA or tacrolimus), CYC, IVIG, MMF, RTX or TNFi, or (2) changing therapy to one of these medications. However, there was no consensus on which specific sequence of medications would be preferred. In order to reflect this uncertainty, the medications or medication classes are simply listed alphabetically (Table 4).

#### Developing a treatment strategy

**Fig. 1:**
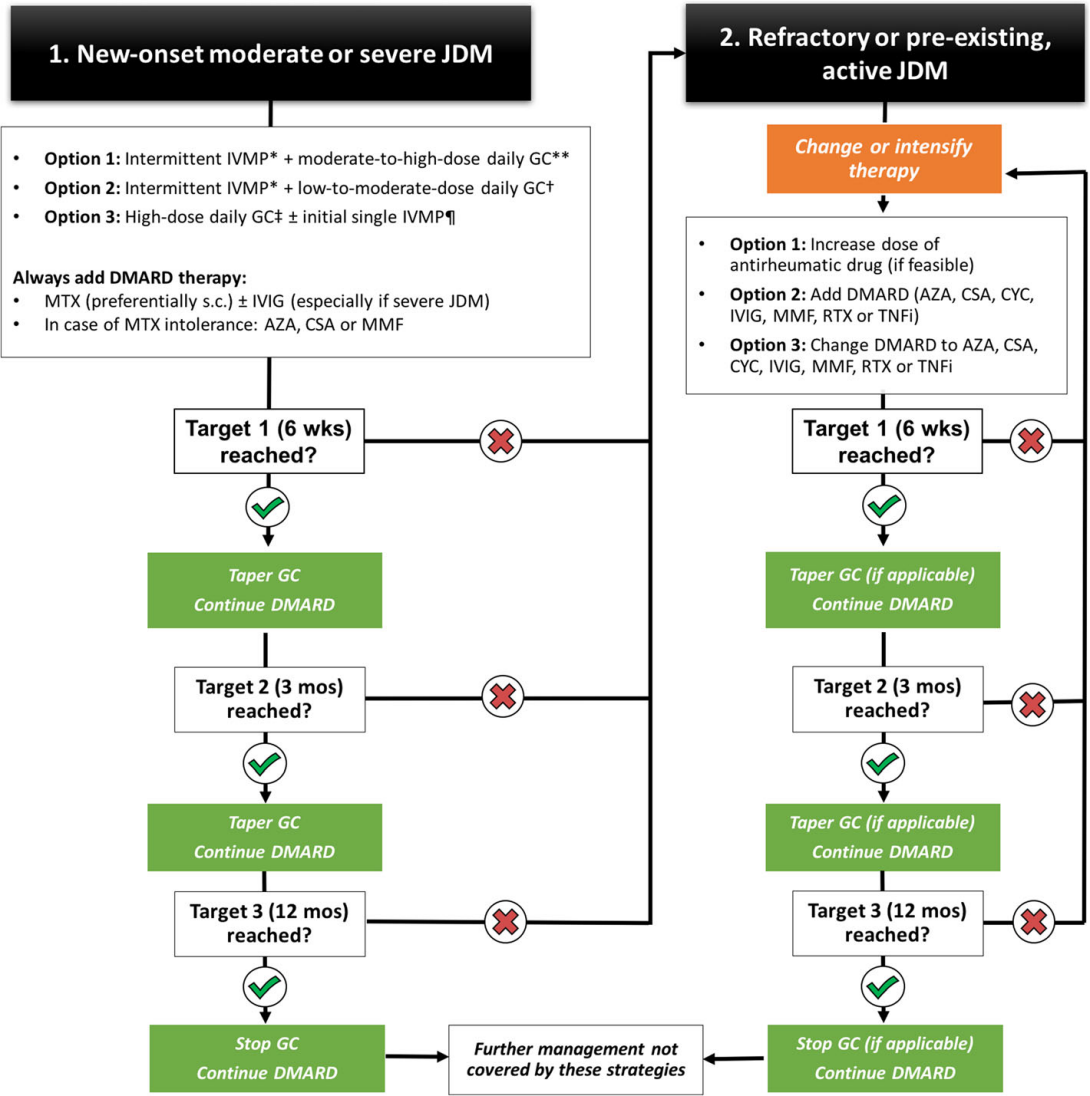
Treatment strategies in juvenile dermatomyositis (JDM). Patients with new-onset active JDM are treated with 1 of 3 glucocorticoid regimens and always receive an additional disease-modifying antirheumatic drug, preferably methotrexate with or without intravenous immune globulins. If the predefined treatment targets are achieved, glucocorticoids are tapered according to a scheduled outlined in Table 3. If treatment targets are not reached, a modification in therapy is needed, i.e. either addition of a new therapy or switching therapy. There was no consensus as to a specific sequence of changes in therapy. Patients may also be treated according to these strategies if they have pre-existing, active JDM (see right side of this figure). *Repeated cycles of intravenous methylprednisolone 20–30 mg/kg (max. 1000 mg) daily for 3–5 days in a row. **Prednisone equivalent 0.5–2 mg/kg (max. 80 mg) daily. †Prednisone equivalent 0.2–0.5 mg/kg daily. ‡Prednisone equivalent 2 mg/kg (max. 80 mg) daily. ¶Single cycle of intravenous methylprednisolone 20–30 mg/kg (max. 1000 mg) daily for 3–5 days. Abbreviations: AZA, azathioprin; CSA, cyclosporin A; CYC, cyclophosphamide; DMARD, disease-modifying antirheumatic drug; GC, glucocorticoids; IVIG, intravenous immune globulins; IVMP, intravenous methylprednisolone pulse; JDM, juvenile dermatomyositis; MTX, methotrexate; MMF, mycophenolate mofetil; RTX, rituximab; s.c., subcutaneously; TNFi, tumor necrosis factor inhibitor

The statements developed are condensed in Fig. 1. Patients with JDM may enter the treatment strategies outlined before either with new-onset disease or with existing long-standing disease. Patients with new-onset JDM are treated with one of the glucocorticoid regimens and a DMARD, preferentially MTX, whereas treatment with existing and active JDM may enter the strategies later. An essential part of the treatment strategies is the treat-to-target concept, i.e. the need for frequent monitoring of disease activity and adjustment of treatment if treatment targets are not reached.

## Discussion

Based on data on the current clinical practice in Germany we defined consensus-based statements and strategies supporting the diagnosis and the management of JDM. The expert panel consisted both of pediatric neurologist and rheumatologists with all of them having practical experience in managing JDM. The group supports the use of “adapted” Bohan and Peter criteria for the diagnosis of JDM, including the use of specific MRI findings as a Bohan and Peter “equivalent” criterion, similar to what others have proposed in the past [2]. There are a variety of diagnostic tests that are frequently employed in Germany to assist in the diagnosis and assessment of patients with JDM, and therefore are represented here. More recent developments, such as the discovery of myositis-specific antibodies (MSAs) that may be rather specific to JDM when compared to adult DM are also included, and it is felt that these antibodies may have diagnostic and prognostic value [36–39]. We defined, to our knowledge for the first time, an explicit treat-to-target strategy for the management of JDM using the recently developed ACR/EULAR response criteria indicating absent, minimal, moderate or major improvement for formal assessment of treatment response [35]. Notably, others have shown that aggressive therapy of JDM using treatment targets may lead to improved outcome [8]. Similarly, the North American CARRA plans guide treatment decisions based on whether patients are unchanged, worsening, or experiencing medication side effects or disease complications [17]. The European SHARE recommendations focus on whether improvement is present or absent to guide treatment decisions but does not precisely delineate time frames for improvement [16]. The treat-to-target concept focusses on strategy rather than on specific treatment modalities which, for example, has been proven superior to conventional therapy in rheumatoid arthritis [40]. While the overall goal of therapy was defined as clinical inactive disease, we did not define this further during the consensus conference. However, we think that it would be reasonable to apply the validated set of criteria by either IMACS or PRINTO [41, 42].

Since there is a relative lack of data from randomized controlled clinical trials (RCTs), and since it is unlikely that large-scale RCTs for JDM will take place, one possible solution to gather more information on the efficacy or inefficacy of various treatments may be to collect “real life” data on disease outcome, i.e. to pursue comparative effectiveness research [43]. An important prerequisite for comparative effectiveness research is the application of uniform and harmonized treatments. Therefore, the harmonization of existing clinical practice has been an important goal for this project. Furthermore, the regular collection of a dataset in patients with JDM is of utmost importance to allow comparison of clinical outcomes, not only nationally but also internationally. Consequently, our statements include the minimal dataset agreed upon by international experts [29].

These harmonized consensus strategies overall fit rather well into the above-mentioned framework set up by the CARRA consensus treatment plans and the SHARE recommendations which had been developed previously or simultaneously, respectively. However, there are important differences in clinical practice in Germany when compared to this framework. For example, practitioners in Germany have a strong preference to choose a glucocorticoid regimen consisting of ongoing intermittent IVMP therapy in combination with low-to-moderate dose glucocorticoids instead of conventional daily high-dose glucocorticoid regimens [19]. There is surprisingly little data available on this important question. Proponents of intermittent IVMP pulse therapy suspect similar efficacy to high-dose daily glucocorticoids but less adverse effects, whereas other data suggest it may be less efficacious [7, 44, 45]. However, there are no definitive data that demonstrate superiority of one glucocorticoid regimen over another. Regarding treatment choices in case of severe or refractory JDM, there is a strong preference for IVIG, similar what is outlined in the CARRA treatment plans. Finally, the group agrees that patients with JDM should be managed in centers with an expertise in JDM. Furthermore, it is of course assumed that treatment decisions should always be shared between patients, families and providers. Since centers participating in the final consensus meeting manage more than half of all registered patients with JDM in Germany, it can be assumed that the consensus reached here is broadly generalizable in Germany. We have carefully worded the statements so that they are not confused with treatment guidelines or strict treatment recommendations.

There are several limitations to the PRO-KIND consensus strategies for JDM. First, the concepts outlined here may not represent the optimal way to manage JDM but rather represent current clinical practice among JDM experts in Germany and are based on a low level of evidence. Second, the statements and resulting strategies do not represent treatment protocols or “To Do”-lists. Management of patients with JDM will remain highly variable. Third, the field is constantly evolving: Some MSAs that were only recently reported in children, for example, anti-3-hydroxy-3-methylglutaryl-coenzyme A reductase (HMGCR) antibodies, are not included in our list but may be useful to determine in some patients, especially in case of refractory myositis [37, 46–48]. Fourth, some of the statements are rather vague, for example tapering of glucocorticoids, the choice of DMARD therapy beyond MTX, DMARD dosing regimens and the definitions of clinical inactive disease or disease flare. This is in part due to the difficulties achieving consensus on more precise statements which, in turn, results from the lack of convincing evidence for the preference of one option over another. We attempt to circumnavigate these issues by the implementation of a stringent treat-to-target strategy as outlined above. Fifth, many of the treatment options outlined in our strategies are not legally approved for the treatment of JDM, including the standard treatment with MTX. Therefore, treatment choices for individual patients may need to be adjusted based on local regulations for the off-label use of certain drugs. Sixth, some of the treatment options may not be widely available, again either due to regulation or due to cost.

## Conclusions

In summary, we have developed consensus-based strategies for the diagnosis, monitoring and treatment of JDM by the harmonization of current clinical practice with the overarching goal is to improve the outcome of patients with JDM. Concerning therapy, we are placing an emphasis on a treat-to-target strategy using internationally accepted improvement criteria rather than on individual medications, fitting well into existing frameworks. As others have done previously, we define a minimal dataset to regularly collect in patients with JDM in order to allow comparative effectiveness research. We conclude that, due to the rarity of the condition, international collaboration will be critical in order to improve the outcome of patients with JDM. We believe that this work will add to the armamentarium of providers managing patients with JDM worldwide.
